# Polysaccharide Hydrogels Doped with MXenes for Possible Biomedical Applications

**DOI:** 10.3390/molecules31010148

**Published:** 2026-01-01

**Authors:** Katarzyna Suchorowiec, Justyna Kasznik, Anastasiia Stepura, Mária Omastová, Kinga Pielichowska

**Affiliations:** 1 Faculty of Materials Science and Ceramics, AGH University of Krakow, Al. Mickiewicza 30, 30-059 Krakow, Poland; 2 Polymer Institute of Slovak Academy of Sciences, Dúbravská Cesta 9, 84541 Bratislava, Slovakia

**Keywords:** hydrogels, MXene, polysaccharides, gellan gum, sodium alginiate, smart biomaterials

## Abstract

MXenes, a new family of two-dimensional transition-metal carbides and nitrides, have attracted significant interest in biomedicine because of their tunable surface groups and multifunctional properties. Hydrogels, with their three-dimensional polymeric networks rich in water, provide excellent biocompatibility and structural similarity to those of biological tissues. Although synthetic polymer–based MXene hydrogels are well studied, polysaccharide-based systems remain underexplored despite their biodegradability and biomedical relevance. In this work, MXene nanosheets were incorporated into a sodium alginate (ALG)–gellan gum (GG) polymeric blend to develop polysaccharide/MXene hydrogels. Two dehydration approaches, conventional drying and freeze-drying were used to evaluate their influence on the characteristics of the composite, including microstructure, surface roughness, compressive behavior, water states, and thermal stability. Conventionally dried polysaccharide/MXene nanocomposites with 1.0% wt. MXene have reduced the swelling ratio by ~60% and the volume change by 40% compared to polysaccharide blend. Freeze-dried polysaccharide/MXene nanocomposite hydrogels developed a porous, interconnected network, making them promising for applications requiring high surface area, such as adsorption and tissue engineering. In contrast, conventionally dried samples formed compact, smooth structures with improved barrier and mechanical performance. These results demonstrate that the dehydration strategy strongly governs the polymer network architecture, water states, and material functionality, offering pathways to tailor hydrogel modified with MXene for specific biomedical applications.

## 1. Introduction

In recent years, the discovery of two-dimensional (2D) materials like graphene with remarkable mechanical properties has spurred a significant increase in the study of 2D materials, leading to the development of a new subgroup of 2D materials—MXenes [1,2]. MXenes are a relatively new type of 2D material (discovered in the 2011) in form of layers consisting of transitional metal carbides or/and nitrides [3]. They are obtained from MAX (M_n+1_AX_n_) phase precursors which determine their unique way of synthesis and final 2D material properties. *M* stands for the transition metal (Ti, V, Nb, Mo), *A* is associated with an element of the 13 or 14 group, *X* comes from the carbon or nitrogen, and n is the number in range from 1 to 4 [4]. After the etching process, the MXenes chemical formula can be formulated as M_n+1_X_n_T_x,_ where T_x_ is associated with different termination groups such as –OH, =O, –Cl or –F. The termination groups depend on the synthesis method and its efficiency. The termination groups are also responsible for the final MXene properties thus the monitoring of the synthesis process is crucial for further incorporation and usage as nanoadditives in composites [5]. The wide range of termination groups reflect the future perspectives of applications such as electronics [6], sensors [7], photoconversion [8] or biomedical applications [9]. Importantly, the intrinsic properties of MXenes can be finely tuned by manipulating the choice of M and X elements, as well as by engineering their surface terminations, thereby offering a versatile platform for diverse applications. Taking into consideration the biomedical applications MXenes have several advantages. First, their hydrophilic characteristic comes from the functional groups that can facilitate the graft of molecules on their surface [10]. They are formed from elements widely found in living organisms: C, N, H and O. Furthermore, they represent a highly negative zeta potential that enables MXenes to form stable colloidal dispersion both in aqueous and organic solvents [11]. MXenes exhibit strong broadband light absorption and efficient light harvesting in the near-infrared range, spanning both the first and second biological windows. Coupled with their high photothermal conversion efficiency, these materials present considerable potential for advanced biomedical applications, including photothermal therapy and photoacoustic imaging [12]. Due to the large surface area, MXenes could act as an efficient drug or gene carriers [13].

Compared to other 2D materials like graphene or carbon nanotubes, MXenes offer tunable surface chemistry due to the presence of various surface terminations (–OH, –O, –F) that can be controlled during synthesis. This functionalization enables enhanced hydrophilicity, improved interfacial interactions, and better compatibility with polymers or electrolytes. Additionally, MXenes demonstrate enhanced electrochemical properties relative to other two-dimensional carbon-based materials, attributed to their intrinsic metallic conductivity, adjustable surface functional groups, and abundant redox-active sites. Their lamellar architecture facilitates fast ion diffusion and elevated capacitance, surpassing the performance of graphene and carbon nanotubes [14].

The incorporation of MXenes into hydrogel matrices facilitates the development of MXene-based soft materials with tunable properties, while simultaneously improving the intrinsic stability of MXenes, a factor that commonly restricts their broader application while creating multifunctional soft material [15]. Simple processing methods such as lyophilization or conventional drying also enable the fabrication of MXene hydrogel derivatives, such as aerogels, further extending their application potential. Recent years have shown that the field has experienced exponential growth concerning MXene hydrogels and their derivatives in publications [16,17,18,19,20,21,22,23], rendering it an increasingly competitive area of research [24]. In comparison to hydrogels derived from other 2D nanomaterials, such as graphene, transition-metal dichalcogenides, and black phosphorus, MXene-based hydrogels present several distinct advantages. First, their inherent high hydrophilicity ensures excellent dispersion and stability of MXene-derived photodynamic and photothermal agents in physiological environments. Second, the abundant polar terminal groups on MXene surfaces facilitate the straightforward grafting of anticancer drugs [15].

Hydrogels can serve as versatile matrices for smart biomaterials, capable of adjusting their properties in response to stimuli. Hydrogel classification is determined by the source and composition of their polymers, placing them into natural, synthetic, or hybrid groups [25]. Their similarity to human tissues comes from the 3D polymeric network created by the interactions of both chemical (covalent) and physical (hydrophobic, electrostatic) and the ability to absorb water up to 99% by weight without dissolution [26,27]. The high water absorption in hydrogels mirrors the tissue microenvironment, making them a promising candidate in biomedical applications [28]. The stable 3D structure of hydrogels during their swollen state comes from chemical and physical cross-linking points. In chemical cross-linking, a low molecular weight cross-linking agent is introduced into the reaction mixture along with the polymer, leading to covalent bonding between polymer chains [29,30]. In contrast, physically cross-linked hydrogels are stabilized through mechanisms such as physical domain junctions, ionic complexation, hydrophobic interactions, and hydrogen bonding [31,32]. However, when there are no cross-linking points, the 3D structure is disrupted due to the polymer chain and water thermodynamic compatibility and polymer dissolution in water. Moreover, hydrogels are similar to human tissues in terms of mechanical and electrical properties, so they are one of the most widely used materials for artificial muscles, skin, or axons [33,34,35].

Nowadays the development of smart items has influenced other fields leaving a significant mark on the medical and biomedical field. The smartness of biomedical devices comes from their ability to react on time and respond to user-patient needs. Furthermore, the ideal medical device should also be able to change its properties according to the signal/factor they received in order to adapt themselves to changing environments [35,36]. Currently, there are numerous smart medical devices (or medically related) and materials covering the need for patient-orientated therapies which are the essence of modern and future medicine [37]. This could significantly influence the effectiveness of the treatment and help minimize the side effects of invasive therapies. The smart material can react based on external factors caused by changes in light, pH, pressure, temperature, oxygen, or other levels of molecules [35]. However, obtaining smart biomaterial could be challenging. First because of the proper design and functionality leading to desired response, second because of the biocompatibility of those materials; hence, the proper design and composition is needed. The search in elastic/shape adaptive materials became a challenge in the biomaterial field [38].

The most used MXene-hydrogel combination is the composites based on poly(vinyl alcohol) (PVA) and Li et al. [39] reported on MXene/PVA antibacterial hydrogel for the treatment of infected wounds by precisely controlling its structure through a combination of directional freezing and a chemical salting process. Regarding conductive hydrogels Chen et al. [40] presented research on hierarchical fabrication strategy to develop strong, tough and multifunctional conductive hydrogels used in wearable sensors.

Polysaccharides are less common when it comes to incorporation of MXene into the hydrogel. The main polysaccharides used as a hydrogel matrix are gellan gum (GG), sodium alginate (ALG), hyaluronic acid (HA), and chitosan (CHI). He et al. [41] conducted a study on MXene nanosheets introduced into hydrogel based on GG via physical mixing to obtain a photothermal conversion for local drug delivery in tumor treatment, demonstrated the good biocompatibility of the composite, good stability, and effective drug release control. Wu et al. [42] conducted research on ALG-based MXene composite with the addition of zinc ions to obtain an efficient sterilization dedicated for practical antimicrobial wound dressing applications. The composite demonstrated high chemophotothermal ablation of bacteria due to the additional incorporation of zinc ions.

This research aims to present the incorporation of MXene nanosheets into the polymeric blend of ALG and GG. Polysaccharides like ALG and GG are particularly used in biomedical applications because they are known and widely tested in terms of biocompatibility, favorable gelation mechanisms, and ability to form structurally robust hydrogel networks. Both polysaccharides are non-toxic, non-immunogenic, and widely employed in tissue-contacting materials, which support their safe integration with bioactive nanomaterials such as MXenes [43]. Research is focused on the comparison of two different drying methods resulting from the low addition percentage of MXene nanosheets and their influence on the material properties with respect to microstructure, surface roughness, and their behavior under compression. The MXene loadings of 0.1%, 0.2%, 0.5%, and 1.0% (*w*/*w*) were selected to systematically investigate the effect of low-to-moderate MXene incorporation on the composite properties. These values represent a commonly used concentration range in MXene–polymer systems, where even minimal additions can significantly modify their behavior. The chosen range also avoids the high-loading regime (>1–2%) in which MXene sheets tend to aggregate and impair dispersion stability.

As hydrogels are well-known for their water absorption properties which are crucial in terms of biomedical applications, the study also aims to provide comparison of different dehydration methods and their impact on the water states, thermal degradation and mechanical behavior. By addressing the underexplored field of polysaccharide–MXene hydrogels, this work aims to provide new insights into their design and potential biomedical applications.

## 2. Results and Discussion

### 2.1. Scanning Electron Microscopy (SEM)

**Figure 1:**
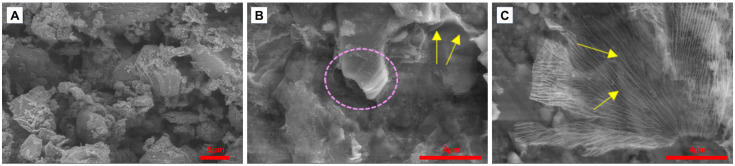
SEM micrographs of (**A**) MAX Phase, (**B**) MXene paste after etching, (**C**) MXene after delamination.

SEM images (Figure 1) illustrate stages of the transformation of the MAX phase into MXene during the fabrication process. The MAX phase in powder form (Figure 1A) has a non-uniform structure, with irregular shapes and different sizes of granules. There are no signs of single layers or sheets. After the etching process (Figure 1B), some accordion-like microstructure (pink circle) can be distinguished where the layers are arranged parallel and adhere to each other. Some singular layers (yellow arrows) could also be seen at this stage. The microimage of MXene after delamination (Figure 1C) shows the MXene where the MXene single layers are stacked one by one forming a tree-like shape. The yellow arrows indicate well-distributed single layers of MXene.

**Figure 2:**
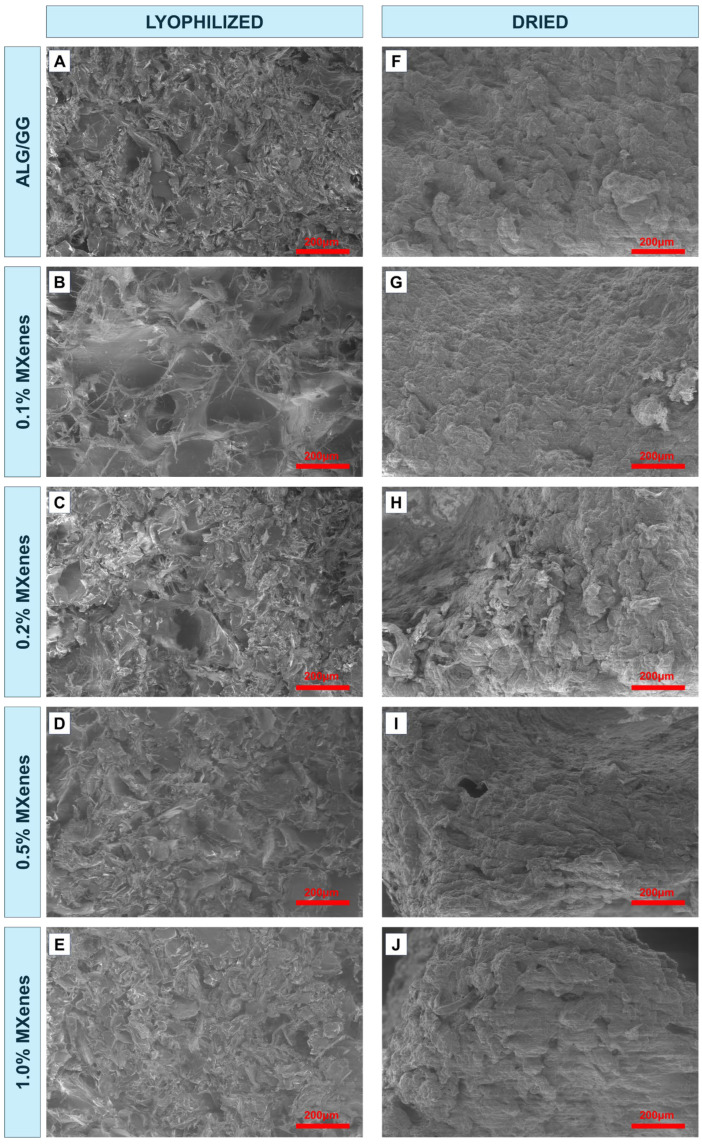
SEM micrographs of lyophilized (**A**) ALG/GG, (**B**) 0.1%, (**C**) 0.2%, (**D**) 0.5%, (**E**) 1.0% MXene xerogels and dried (**F**) ALG/GG, (**G**) 0.1%, (**H**) 0.2%, (**I**) 0.5%, (**J**) 1.0%, MXene xerogels.

SEM images of xerogels (Figure 2) indicate that the dehydration process could have a significant impact on the microstructure of the hydrogels. Freeze-dried samples show higher porosity; the pore shapes are less uniform with sharp flakes, edges, and loosely interwoven network. The structure is more developed and spatial compared to dried samples. Additionally, some thin thread-like parts may be distinguished that could be ascribed to the polysaccharide net created through the cross-linking. At the low percentage MXene sheets do not significantly influence the microstructure but the network is less dense (Figure 2B–D), while 1% MXene sample has a more uniform pores and polysaccharide sheets distribution, but there are less pores (Figure 2E). This could be the result of a higher amount of free water, as water molecules could aggregate onto the ice crystals more easily and are less influenced by the polymer chains, and indicate that the hydrogels with MXenes are less crosslinked. The SEM micrographs could suggest that the pores that have been created during the ice crystal sublimation are interconnected, which could result in better paths for water to be reabsorbed if they would undergo the rehydration process. The microstructure of dried samples (Figure 2F–J) has limited porosity and is more closed and smoother, which is caused by the capillary forces of water evaporating from the sample. There are no sheets or threads because the polysaccharide network has shrunk.

Freeze-dried samples display a highly porous and interconnected microstructure, indicative for enhanced surface area development. Such morphology is advantageous for applications where high surface-to-volume ratios are critical, including adsorption processes and tissue engineering scaffolds. In contrast, conventionally dried samples exhibit a comparatively compact and smooth architecture, which may confer improved mechanical integrity and barrier performance, making them suitable for structural or protective applications.

### 2.2. Optical Digital Microscopy Observations

**Figure 3:**
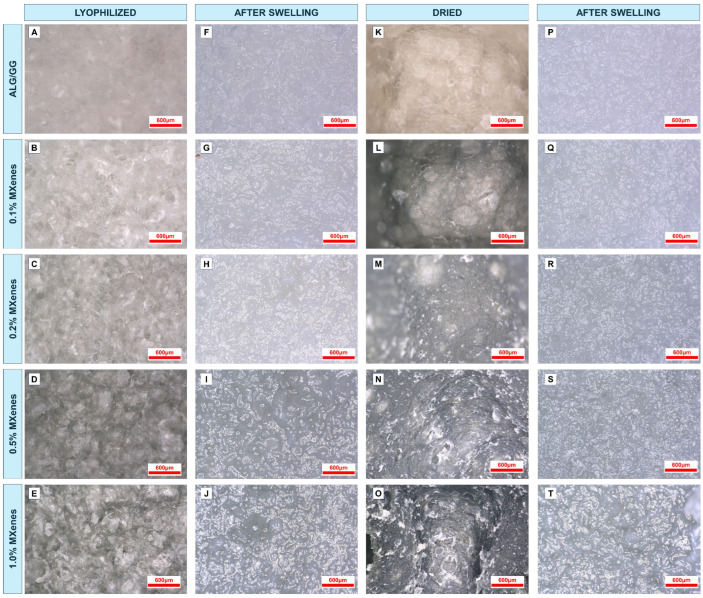
Digital optical microscope photographs of lyophilized hydrogels before (**A**–**E**), after swelling (**F**–**J**) and photographs of dried hydrogels before (**K**–**O**), and after swelling (**P**–**T**).

Optical photographs of lyophilized samples before (Figure 3A–E) and after the swelling (Figure 3F–J) indicate the changes in the material macrostructure as they undergo water absorption process. Freeze-dried hydrogels retain their original (given) shape, the pores are open and well distributed with similar sizes, and they are not collapsed and show their original shape. Additionally, the pore walls are thin and well developed. The ALG/GG sample, which does not contain MXene, is white, whereas with increasing MXene concentration, the color of the material darkens, and its color becomes uniformly grey, which indicates good dispersion of the nanoadditives within the matrix. In the 1.0% MXene sample (Figure 3E) few dark spots were observed in the matrix, which may indicate agglomerated MXene molecules. Samples, with 0.5% and 1.0% of MXenes (Figure 3D,E) exhibited more spherical pores (remained from water removal). After immersion in water original shape of the samples was maintained. The pores that have been previously spotted filled up with water. Only the largest pores are empty because of the cohesion forces of water that have been removed from the surface of the sample by the tissue paper. With a higher percentage of MXenes, there is an increase in number of empty pores. The matrix has increased the thickness of the netlike walls, which indicates that the water is trapped not only within the pores but also within the polymeric chains. Like the xerogels, the sparse agglomerated MXene particles are visible in the 1.0% MXene sample (Figure 3J).

The dried xerogels exhibited a darker color range than the corresponding lyophilized xerogel. The surface is denser; there are only a few pores visible. As the MXene content increased, the samples tended to create a sharper gap on the surface, and the macrostructure was less uniform. The addition of MXenes may lead to higher thermal conductivity and the quicker temperature increase, and thus faster matrix collapse. After the swelling process, the samples returned to their previous shape and there were no signs of surface collapse. This may be caused by the denser matrix and faster water distribution within the polymeric chains.

### 2.3. Surface Roughness

**Figure 4:**
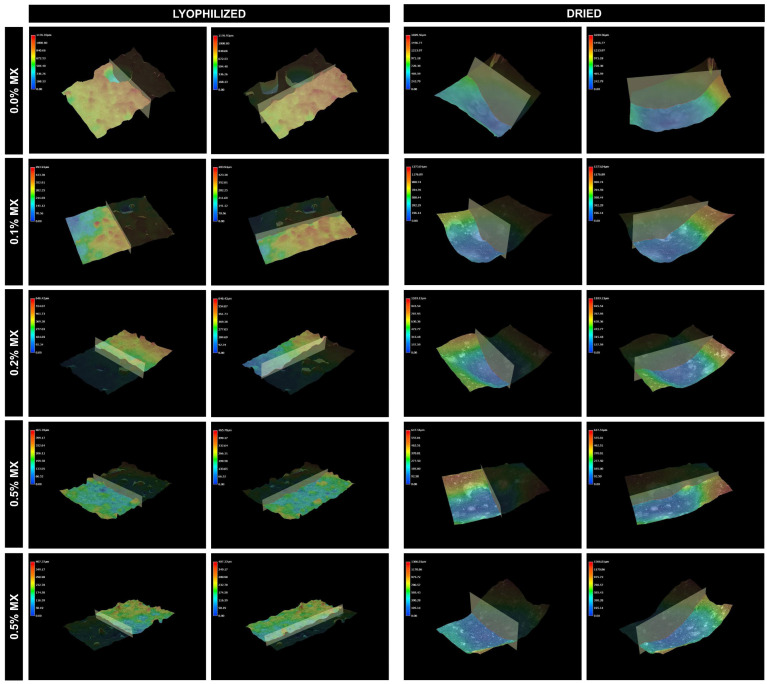
Digital images of 3D surface roughness evaluation of xerogels in perpendicular and parallel axis.

**Figure 5:**
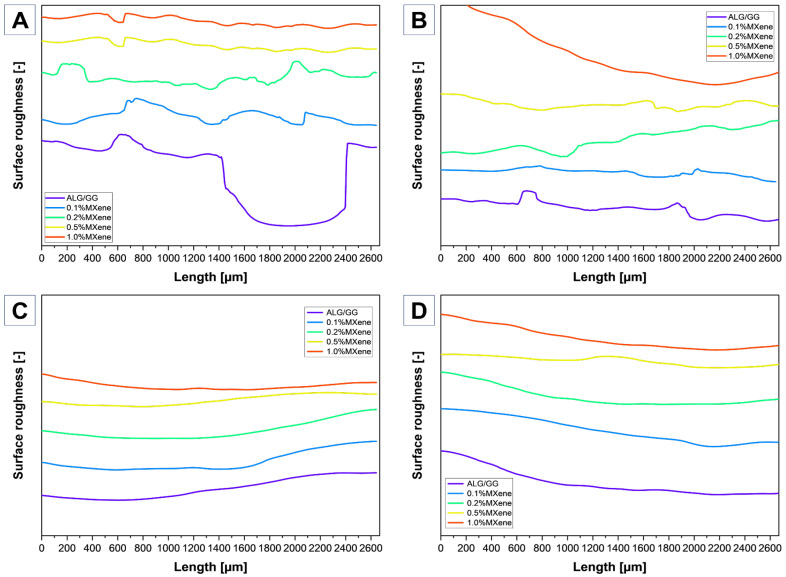
Surface profile from digital microscope 3D scanning technique lyophilized (**A**) perpendicular, (**B**) parallel, dried (**C**) perpendicular, (**D**) parallel.

The surface roughness of the hydrogel material plays a crucial role especially in terms of swelling and dehydration. The rougher surface led to further enhanced water uptake caused by capillary forces and the diffusion of water. In terms of swelling and shape control, the smoother surface allows more unform swelling fulfilling the dimensional stability requirement. The dehydration process is also affected by roughness; rougher surfaces may facilitate faster water evaporation due to increased surface area exposure. When it comes to xerogel fabrication, the lyophilization process led to the obtaining of surface area corresponding to the swollen hydrogel with open pores and a high active surface area. From the 3D scan (Figure 4) the even distribution of surface-open pores could be seen for the samples 0.5% and 1.0% MXene content. With increased MXene addition, the surface profile is more even (Figure 5A,B).

On the other hand Figure 5C,D shows almost even surface without significant fluctuation coming from the oven-drying of hydrogels. One difference could be noticed, the collapsed surface coming from the stage II water evaporation process of weakly bound water, causing network collapse and bigger crater—like surface pores [44].

### 2.4. Equilibrium Degree of Swelling (EDS)

**Figure 6:**
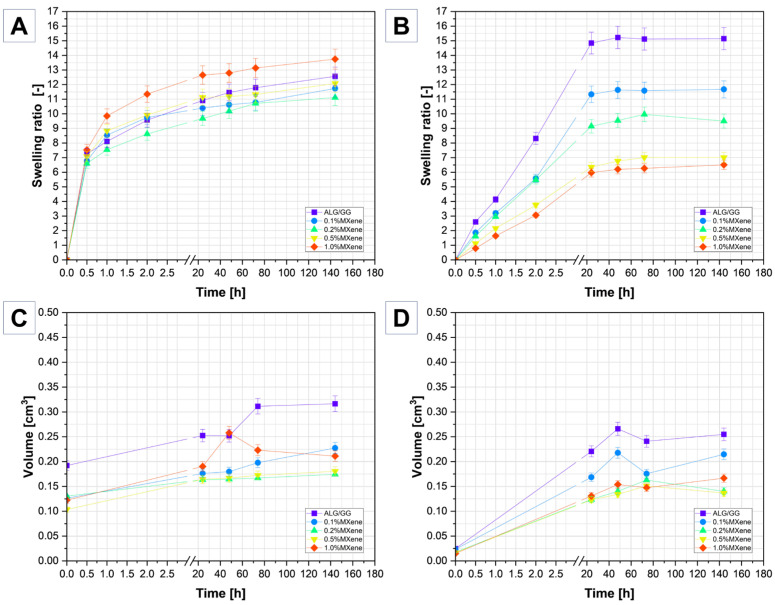
Swelling ratio of (**A**) lyophilized and (**B**) dried samples, volume change of (**C**) lyophilized and (**D**) dried samples.

Figure 6A,B show the water absorption graphs of MXene-modified hydrogels after lyophilization and conventional drying, respectively. Immersing xerogels in deionized water led to the expansion of their network structure and uptake of water, increasing the sample mass. The swelling process performs as follows: after the insertion of the dry hydrogel into the water, at first the polar parts of the polymer chains interact with the H-bound, leading to the water polymer bounding. Consequently, the polymer network swells, exposing its most hydrophobic segments to water, triggering further interactions between the polymer chains and water molecules [45,46]. The swelling process (Figure 6A) of lyophilized samples could be divided into three main parts: I part of water absorption is from zero to 30 min, when the water intake starts rapidly, II part: from 30 min to 1 h water intake starts to slow down, III part from 1 h to 144 h (6 days)—water absorption and intake increase is almost constant. For comparison dried samples swelling process (Figure 6B) could be divided into two main parts: I part of water absorption is from zero to 24 h—the water intake is smaller (compared to the lyophilized samples) and stable during 24 h. II part of swelling from —24 min to 144 h (6 days)—is almost constant with small differences across the measuring gaps. After 144 h all of the samples showed small increase/no change/or small decrease, which indicates that the water absorption process has stopped, which means that hydration forces (coming from the hydrophilic chains of polysaccharides) are counterbalanced by the elastic restoring forces coming from the crosslinked chain. If the cross-linking density is higher, the hydrogel will absorb less water. The forces driving water uptake and retention are balanced by expanding the polymer network until equilibrium is reached, which marks the maximum water absorption capacity of the hydrogel. Therefore, swelling examination could be an indirect method for determining the cross-linking density of a hydrogel, when a swelling ratio (SR) is low, the cross-linking density is high and vice versa. Lyophilized sample with 1.0% of MXenes exhibited the highest SR = 13.75, which is a result of the larger pores created by the less cross-linked polymer network. Additionally, it could be influenced by the Donnan effect of negatively charged GG chain and MXene functional groups, resulting in higher water uptake. The overall EDS value of other samples was also high and oscillated to the 12 without significant correlation with the share of MXene in the material.

Dried samples tended to rehydrate more slowly, and the presence of MXenes decreased the water absorption capacity of the hydrogel which may be caused by the enhanced thermal conductivity of the sample and thus thermal degradation during the drying process. Another reason could be the additional cross-linking that occurred during an increase in temperature and limitation of water absorption; thus, the higher the MXene content, the denser the network. Surface –OH and –O groups can form hydrogen bonds with polar groups in polymer chains. This introduces physical cross-links that reinforce the hydrogel network without the need for additional chemical agents that means that the MXene sheets can act as a crosslinker themselves. In the case of 0.1% and 0.2% wt. MXene samples, the EDS values are similar in both lyophilized and dried samples (~10/~11). In 0.5% and 1.0% wt. MXene samples, the value of SR are almost two times lower (~7). The presence of MXene influences the balance between hydrophilic expansion and hydrophobic contraction during the drying process resulting in a less swellable structure after dehydration. The reduced swelling ratio post-drying in MXene-rich samples indicates that MXenes limits the rehydration capacity, possibly by forming a more compact and interconnected structure during water loss.

The volume change parameter (Figure 6D) showed a high increase in the first 48 h slowing of swelling of the dried samples. Then some of the samples stabilized. The sample with 1.0% MXenes has reached the biggest volume although it has the smallest EDS. This may be caused by the volume that expanded and absorbed water during the swelling process. For lyophilized samples (Figure 6C) the main change (especially in 0.5% and 1.0% of MXenes) is present within 24 h, in most of the samples the percentage increase continues, but the differences are not that big, except for 1.0% MXene sample, where the jump to ~110% is very noticeable, but after that some decrease is also present. This may indicate that the hydrogel expands quickly, but then partially contracts or stabilizes.

### 2.5. Differential Scanning Calorimetry (DSC)

**Figure 7:**
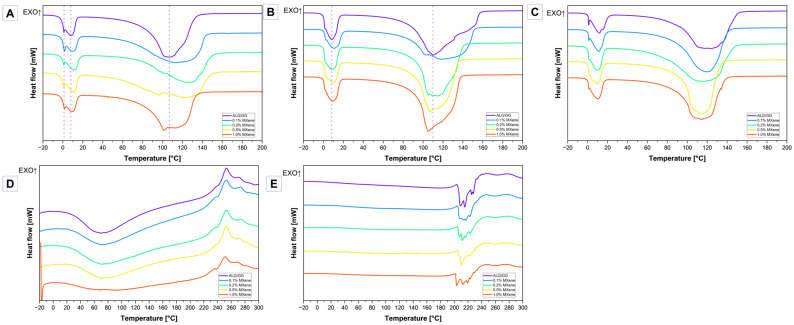
DSC curves of (**A**) fresh crosslinked hydrogels, (**B**) lyophilized xerogels-hydrogels after swelling, (**C**) dried xerogels-hydrogels after swelling, (**D**) lyophilized xerogels, (**E**) dried xerogels.

Figure 7A shows the DSC heating curves of hydrogels with different MXene content after the crosslinking process. The first endothermic peak (from −8 to 17 °C) is associated with freezing bound water (sharp part of the first endothermic peak), and free water (broad part of the first endothermic peak). Free water is water similar to that in the bulk state and involves only water-water interactions, so the broad peak temperature is close to 1 °C. On the other hand, freezing bound water not only have water-water interactions, but also it can interact with the polymer chain through a weak van—der—Waals bond [47]. The DSC curves showed that with the higher amount of MXenes in the hydrogel matrix, the temperature corresponding to the broad part of the first endothermic peak is higher (10/11 °C compared to 8 °C from pure ALG/GG) and the share of the peak is larger, suggesting bigger amount of free water. There is also a third type of water in hydrogels —non—freezing water that is strongly bound to the polymer matrix mainly through hydrogen bonds, which is why it does not create interactions between itself and there are no solid-liquid phase transitions that can be observed by DSC study. The unusual behavior of water in polymer solutions is explained by capillary condensation, where polymer chains restrict the formation of water clusters [47]. The second endothermic peak on the DSC curves (Figure 7A) corresponds to the evaporation of water, it begins at about 50–80 °C (depending on the type of sample) and ends at about 120–150 °C. The pure ALG/GG DSC curve exhibits only one almost symmetric, while the curves of hydrogels with MXenes addition change significantly. The temperature range for MXene-modified cross-linked hydrogels is broader (Table 1). Additionally, as the MXene content increases, two distinct peaks begin to emerge more clearly. Samples with concentrations of 0.1%, 0.2% and 0.5% show a significant shift of this transformation compared to the control sample (Figure 7A). The peak for hydrogel with 1.0% MXene also shifted, but to a lesser extent. Similarly to the melting process, the MXene nanoadditive prolongs the time of water evaporation from the hydrogels.

Figure 7D,E show DSC curves of xerogels after lyophilization and conventional drying process. There are some thermal effects that are observed in dehydrated hydrogels. The lyophilized sample curves (Figure 7D) show an endothermic peak ~70 °C (probably some remaining water caused humidity and hydrophilicity of the material), and it becomes less sharp and wide as the MXene content increases. The exothermic peak may be ascribed to the start of the degradation of polymeric matrix and decarboxylation reaction of carboxyl groups. The DSC curves of the dried xerogels do not exhibit any endothermic thermal effect ~100 °C (Figure 7E) but exhibit an endothermic thermal effect with several sub-peaks (200–220 °C) followed by the exothermic thermal effect (230–300 °C). These effects could also be ascribed to the reactions occurring within the thermal decomposition processes of the polysaccharide matrix.

There are no tendencies ascribed to the presence of MXene content in xerogels and their thermal performance. Throughout the analysed temperature range for all material types, no indications of the glass transition were observed. This may be due to the strong hydrogen bonding both within and between molecules, as well as the rigid, dry structure. Figure 7B,C show the DSC curves of hydrogels at the ES. The curves representing swollen hydrogel exhibited same thermal effects, resulting in endothermic peaks— first one from water melting and second— from water evaporation. Hydrogels that underwent lyophilization before the swelling process (Figure 7B) have a melting peak (8 °C) that differ, mainly in shape, from the “fresh” crosslinked hydrogel resulting in the absence of the double peak. The peak that corresponds to free water is wider and most probably has overlapped the freezing bound water sharp peak. The increase in MXene content did not result in the any significant change in peak shape, only a small shift toward higher temperatures (10/11 °C) could be observed (Table 2) as well as a wider temperature range of the meltin process. The water evaporation peak is sharper, indicating that after the first dehydration and swelling process, water could be removed more easily from the sample. Hydrogels that underwent conventional drying before the swelling process (Figure 7C) have exhibited double peak in first endothermic effect, but the peak from the melting of bound water is not as visible as it has been in the “freshly” crosslinked hydrogels. Additionally with the increasing addition of MXenes the division between two peeks in the melting process is even less significant. Similar to the case of lyophilized swollen samples, the evaporation peaks are sharper.

### 2.6. Thermogravimetric Analysis (TG)

**Figure 8:**
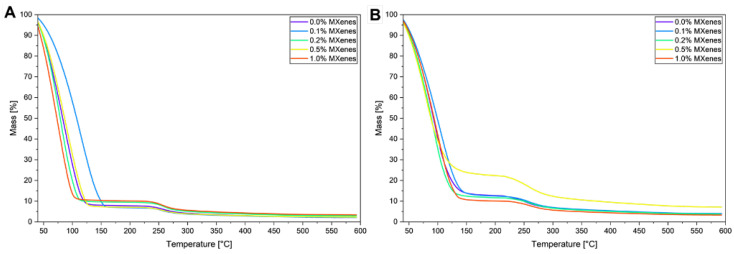
TG curves of (**A**) lyophilized swollen hydrogels, (**B**) dried swollen hydrogels.

In TG (Figure 8) curves there is mass loss hydrogels (~40 °C to 160 °C) ascribed to water loose from. The dehydration of hydrogels is mainly attributed to hydrogen bounding that is associated with nonfreezing bound water. At the first stage of dehydration, the free water evaporates, which can cause shrinkage in the polymer network [48]. The second stage includes the evaporation of the water that is weakly bound to the polymeric chain. In this stage water rapidly evaporates from hydrogel causing network collapse. In the third and last stage of dehydration, water bounded with hydrogen bonds, evaporates from the dried hydrogel slowly [49].

With an increasing percentage of MXenes in the hydrogel, for samples with a concentration above 0.5%, a shift of the mass loss rate to lower temperatures is observed (Table 3). On the other hand, low concentrations of MXene at the level of 0.1% cause a shift of mass loss toward higher temperatures. This effect may be attributed to the additional enhanced cross-linking density coming from small amounts of MXenes (but too small to increase thermal conductivity). Additional cross-linking forms tighter polymer chain traps inhibiting water molecules movements and evaporation, suggesting higher share water that interact with the polymer chain through hydrogen-bound [50]. Higher amounts of MXenes in the hydrogels facilitate heat transportation in the material, causing a decrease in thermal stability or the formation of weak points in the matrix, accelerating thermal degradation [51].

The second mass loss may be observed in the temperature range 210–300 °C and is due to the decomposition of the polysaccharide chain. The shift to higher temperatures in 0.5% and 1.0% samples comes from the higher share of nanoadditives that are more resistant to thermal degradation.

### 2.7. Water States in Hydrogels

**Figure 9:**
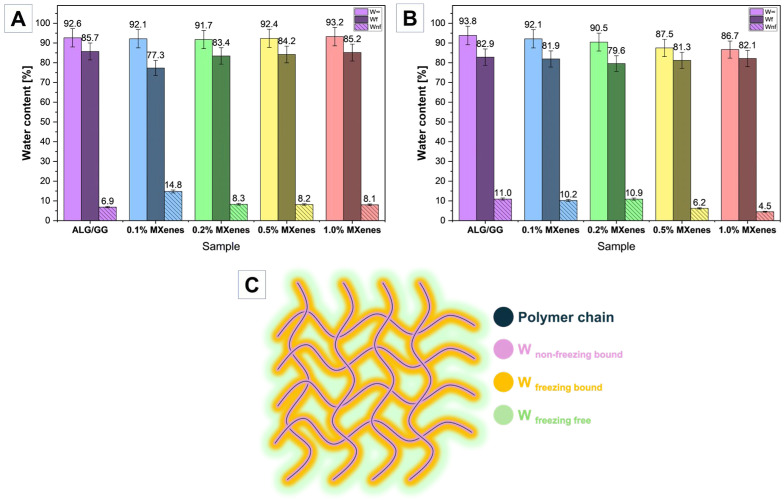
Water states in hydrogels after (**A**) lyophilization, (**B**) drying, and (**C**) schematic illustration of water states within polymer chains. In (**A**,**B**): W_∞_—water content at the EDS (basic color), W_f_—freezing water (darker shade), W_nf_—non-freezing water (hatched).

Monitoring the water states in hydrogels is crucial for the design and tailoring of material properties for the final applications. Different water states could significantly influence the hydrogel behavior in terms of mechanical properties, water absorption, desorption, and ion and chemical substances transportation that could also influence material bioactivity. The lyophilized samples (Figure 9A) exhibited similar values of water content ranging from 91 to 93%, with the highest value for 1.0% MXene sample. The share for the W_nf_ is higher for MXene modified hydrogels, reaching its highest value for 0.1% MXene content ~15% which is almost two times higher compared to pure ALG/GG (~7%). This could confirm that MXenes presence could provide additional water-matrix interaction resulting in hydrogen bonding and changing water behavior in the hydrogel.

In case of oven-dried samples, the water states share after dehydration showed different correlation. With the increase of the MXenes content, the overall water content in the hydrogel decreases. The freezing water content remains at a similar value from 80 to83% without direct correlation. The non-freezing bound water content in dried and thenrehydrated ALG/GG samples is ~3% higher compared to the lyophilized. That may be caused by the polymer chain aggregation and thus stronger polymer chain-water interactions. Lyophilized samples, due to the porous structure that has been maintained, have more water adsorption paths and thus more free or loosely bound water can be absorbed, leading to a lower fraction of non-freezing bound water. A significant decrease in non-freezing bound water content was observed for 0.5% and especially 1.0% MXenes sample, where the value is almost two times smaller reaching only 4.5%. This may be attributed to the additional cross-linking that caused the network and limited chain-water reaction.

### 2.8. Mechanical Characterization

**Figure 10:**
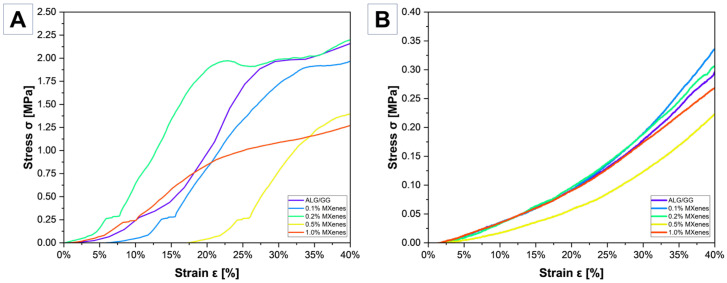
Stress-strain curves for compression tests: (**A**) xerogoels (lyophilized), (**B**) wet hydrogels.

The curves in Figure 10A have characteristic irregularities, suggesting the fragility and porous structure of the freeze-dried samples. These irregularities are typical for brittle, foam-like xerogels in which compression causes progressive pore-wall failure. When the porous, freeze-dried network is loaded, individual cell walls buckle or fracture, producing characteristic drops or steps in the stress–strain curve. The stress-strain curve for ALG/GG shows numerous irregularities, and at a deformation of about 28% there is a sudden step, which may result from the porosity of the sample. This behavior confirms that the mechanical response is dominated by pore-wall instability. Such events are typical for brittle, porous biomaterials, where crack propagation occurs stepwise manner and is followed by partial densification of the structure as the pores collapse. Under the influence of the applied force, the structure above the pores could have cracked locally, which caused a visible step on the graph. The sample containing 0.2% MXene exhibits the highest compressive strength. Its curve shows an initially linear elastic region, indicating a uniform load transfer through the pore walls, followed by a transition to a plastic or densification zone at approximately 1.8 MPa. This behavior suggests that at 0.2% concentration, MXene nanosheets are sufficiently well dispersed to reinforce the xerogel structure by acting as load-bearing fillers and forming additional physical crosslinks (e.g., hydrogen bonding or van der Waals interactions) with the polysaccharide chains. As a result, the pore walls exhibit increased stiffness and resistance to failure.

A similar tendency occurs for the 0% and 0.1% MXene samples. Their relatively high strength indicates that the intrinsic stiffness of the polysaccharide network already provides substantial resistance to compression; therefore, small additions of MXene produce only marginal changes in the porous architecture.

On the contrary, the 0.5% MXene sample shows the weakest compressive strength, and plastic deformation occurs already in the initial loading stage. Higher concentrations of MXene (0.5% and 1%) lead to a decrease in strength, which can be attributed to two structural effects: (i) nanosheet aggregation that creates stress-concentrating defects and weak interfacial regions, and (ii) disturbance of ice-crystal growth during freezing, producing a less uniform and more heterogeneous pore structure. Both effects impair the continuity and load bearing capacity of the pore walls, resulting in premature collapse under compression. Thus, MXene may enhance the mechanical properties of the xerogels only within an optimal concentration window; beyond this range, the disadvantages of aggregation outweigh reinforcement benefits.

The results indicate that the addition of MXene to the hydrogels improves its mechanical properties, with the highest reinforcement observed for 0.2% MXene. However, the use of higher concentrations of MXene (0.5% and 1%) leads to a decrease in strength because increasing the MXenes content affects the mechanical properties, but not in a linear manner—differences in the deformation pattern are visible depending on the concentration of MXene. Samples with 0.1% and 0% MXenes show the highest compressive strength, suggesting that MXenes strengthen the material structure, but may also cause higher stiffness.

In Figure 10B all curves show non-linear behavior—applying a small force causes significant deformation of the samples. This is due to the structure of the hydrogels and the presence of water. At low compressive loads, all samples undergo substantial deformation with minimal resistance. This initial soft regime reflects the high mobility of polymer chains in the fully hydrated state, where water acts as a strong plasticizer by reducing intermolecular interactions and loosening the effective crosslink density. In this region, the deformation is governed by chain reorientation, water redistribution, and viscoelastic relaxation of the hydrogel matrix.

As the pressure force is increased, the polymer chains are reorganised, which leads to the hardening of the material and the need to apply a higher force to cause further deformation. This stiffening corresponds to the progressive expulsion of free water from the pores and to the closer packing of the polymer network. During this stage, the mobility of chains decreases, load transfer between network junctions becomes more efficient, and the hydrogel begins to resist further deformation more strongly. The curves in Figure 10B indicate more elastic and viscoelastic behavior of the hydrogels. All samples show a similar course. In contrast, the 0.5% MXene sample exhibits the lowest compressive strength in the hydrated state. The loss of mechanical integrity at higher concentrations of MXene can be attributed to nanosheet aggregation and disruption of polymer–polymer interactions, which weaken the hydrogel network. Excess MXene can also alter the water distribution and increase local swelling, further reducing the effective cross-link density and compromising the load transfer. MXenes of 0.1% and 0.2% improve mechanical strength compared to pure ALG/GG, suggesting an optimal concentration to maintain a balance between flexibility and strength, resulting from improvement in mechanical properties. This is a similar behavior as that observed by Hou et al. [52], what also indicates a decrease in mechanical properties when the MXene content has reached a higher value but an increase at lower percentages. Despite differences in stiffness, all samples recovered their original shape after compression, confirming their predominantly elastic and viscoelastic nature in the hydrated state. The ability to recover after deformation also supports the conclusion that water governs the mechanical response: the presence of free and loosely bound water facilitates reversible chain motion. A higher compressive modulus generally resulted in less water uptake. In addition to the cross-linking density, the formation of ionic bonds and the hydrophilicity of the network influence swelling and thus mechanical behaviour.

## 3. Materials and Methods

### 3.1. Materials

The MAX phase Ti_3_AlC_2_ 40 microns was purchased from Carbon-Ukraine (Y-Carbon) LLC (Kiev, Ukraine). For in situ HF etching LiF (≥99%, 99.9%, and HCl (35–37% (aq) from Sigma-Aldrich, Darmstadt, Germany) was used. Delamination process of MXenes was supported by LiCl (≥99.9%, Sigma-Aldrich). For the preparation of hydrogels ALG (ACROS Organics, Geel, Belgium) and GG (Sigma-Aldrich, Germany) was used together with the cross-linking agent CaCl_2_ (99.9%, POCH, Avantor Performance Materials, Gliwice, Poland).

### 3.2. Material Preparation

#### 3.2.1. Preparation of MXene Solution

**Figure 11:**
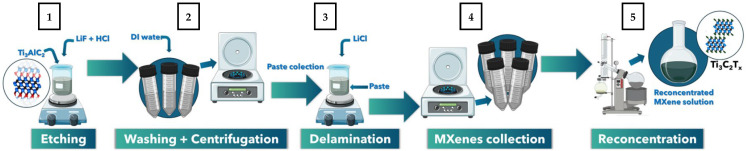
Preparation scheme for MXene solution.

MXene layers were prepared through MILD (Minimally Intensive Layer Delamination) method. First, the MAX phase was etched through the in situ reaction. The etching (1) process started from placing a beaker with cold water and ice on the magnetic stirrer 300 rpm. 78 mL of 37% HCl was poured into the bottle and closed maintaining a funnel for insertion of following substrates. 5 g of LiF was slowly added to the bottle containing HCl through the funnel to obtain the etching solution. After 30 min, 5 g Ti_3_AlC_2_ MAX phase was slowly added to the mixture. The reaction temperature was maintained in the range ~15–20 °C. After all of the MAX phase was added to the etching solution, the mixture was left for 24 h on the magnetic stirrer set up for 300 rpm and temperature of 35 °C. Subsequently, the obtained multilayer MXene paste was washed through the centrifugation process (2) to reach a supernatant of pH ~5. Then, the multilayered MXene paste was collected. The delamination process (3) was carried out with the addition of LiCl in weight proportion (1:1) as an interlayer delamination agent. The mixture was left in the closed beaker on the magnetic stirrer (300 rpm) in the water bath (35 °C) for 24 h. The MXene collection process (4) was performed by numerous centrifugation cycles and collection of the MXene supernant. Then, the MXene colloidal solution was reconcentrated with the rotary evaporator. The concentration of the reconcentrated solution (5) was determined to be 2,3 mg/mL. The process is schematically presented in Figure 11.

#### 3.2.2. Preparation of ALG-GG-MX Hydrogels

**Figure 12:**
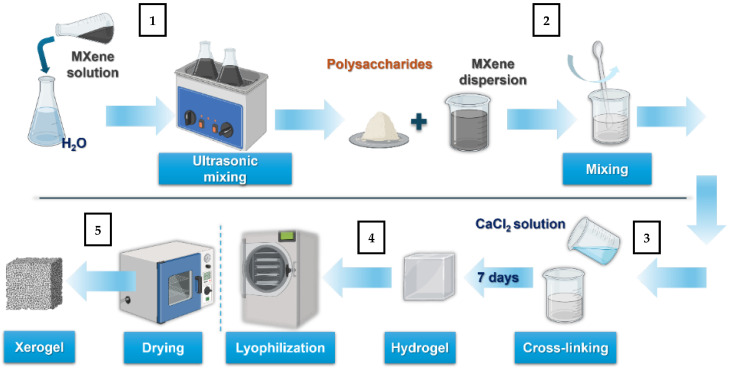
Preparation scheme for Hydrogel/MXene hydrogels and xerogels.

MXene solution was added to pure water and left in 20 °C ultrasonic bath for 15 min (1). The amount of MXene reconcentrated solution added to the water corresponded to the required mass of MXene of 0.1%, 0.2%, 0.5%, and 1%, in the final hydrogels. 8 wt.% polysaccharide mixture was prepared through intensive mixing of water/MXene dispersion with 1:4 (2), ALG:GG mass per unit mixture. The solution was cross-linked with a 0.075 M CaCl_2_ solution (3). The material was left for seven days for crosslinking. The hydrogel and xerogels preparation is shown in Figure 12.

#### 3.2.3. Preparation of ALG-GG-MX Xerogels

In this study, two different methods of material dehydration were used: lyophilization (4) and drying (5). For the lyophilization the hydrogels were cut to the cubic shape (5 × 5 × 5mm) frozen at −80 °C for 24 h and subsequently freeze-dried for 24 h (temp −40 °C and 0.055 mbar/7 Pa). The drying process was carried out on cubic samples of the same dimensions for 24 h at 60 °C using laboratory dryer. The drying parameter 60 °C for 24 h have been selected to ensure the full removal of water from the hydrogel matrix, and avoid intense water evaporation at higher temperatures, but at the same time to prevent the degradation of the matrix and the additive.

### 3.3. Methods (Characterization)

The microstructure of the MAX phase, the MXene paste and MXene after delamination together with the microstructure of the xerogels were studied by scanning electron microscopy (SEM) (Apreo 2S low vac. Waltham, MA, USA) to evaluate the changes in microstructure at each step of the MXene procedure and changes caused by the dehydration process of hydrogels and different additions of MXene. Observations were made under high vacuum conditions (50 Pa) using a secondary electron ETD detector with an acceleration voltage of 10 kV.

Optical microscopy, performed on a Keyence VHX-900F instrument (Mechelen, Belgium), was used to evaluate the morphology of the samples before and after swelling process and also to determine the surface roughness of xerogels using the 3D scanning technique.

The swelling process was performed on cubic (5 mm × 5 mm × 5 mm) freeze-dried and dried samples. Before starting the test, the xerogels were weighed and their dimensions were measured. The samples were placed in 50 mL of distilled water, the same procedure was followed for all freeze-dried and dried samples, and then they were placed in an incubator at 20 °C. The mass measurements were taken after draining the samples softly on filter paper in 0.5; 1; 2; 24; 48; 72; 144 h time gaps. After the measurement, they were placed back in distilled water at 20 °C. The measurement was completed when the mass of the samples began to decrease, which indicated the beginning of degradation of the hydrogel matrix. After fixed time, the dimensions of the samples were measured to determine the change in weight and volume of the hydrogels after the swelling process.

From the swelling process, SD (swelling degree) was calculated according to Equation (1), where SD is swelling degree, is $W_{h}$ sample weight at the time and $W_{x}$. is xerogel weight.

(1) $$SD=\frac{W_{h}-W_{x}}{W_{x}}$$

Additionally, volume change of the samples $V_{c}$ was calculated according to Equation (2), where $V_{h}$ is sample volume at the time, and $V_{h}$ is xerogel volume.

(2) $$V_{c}=\frac{V_{h}-V_{h}}{V_{x}}\cdot 100\%$$

The thermal properties of the materials (xerogels, hydrogels, swollen hydrogels) were examined using differential scanning calorimeter DSC1 (Mettler Toledo, Greifensee, Switzerland) with STARe System software (V16.40). Measurements were performed in two steps cooling and heating. For cooling the temperature range was 25 °C to −20 °C with cooling rate of 10 °C/min and an atmosphere of N_2_ with 30 mL/min flow. For heating the temperature range was −20 °C to 300 °C with a heating rate of 10 °C/min and an atmosphere of N_2_ with 30 mL/min flow. The ~6 mg samples were placed in pierced aluminum pans. The DSC analysis was performed to determine the thermal properties ascribed to states of water in hydrogels and compare them among four different types of samples, crosslinked, lyophilized, dried, and swollen after lyophilization and swollen after drying.

The states of water were calculated using the EDS (equilibrium degree of swelling) and heat of fusion from the DSC results of swollen samples according to Equations (3)–(5). Equation (3) was used to determine the percentage of $W_{f}$ freezing water (free water and freezing bound water) where $∆H_{endo}$ is the heat of fusion of the first endothermic peak on the DSC curve corresponding to the melting of water in hydrogel. The $∆H_{w}$ is the heat of fusion of pure water (333.33 J/g) [53]. To calculate $W_{nf}$ (non-freezing bound water) in swollen hydrogels Equation (5) was used where $W_{\infty }$ is the water at the EDS according to the Equation (4).

(3) $$W_{f}=\frac{∆H_{endo}}{∆H_{w}}\cdot 100\%$$

(4) $$W_{\infty \;}\left(\%\right)=\frac{{EDS}_{max}}{{EDS}_{max}+1}\cdot 100\%$$

(5) $$W_{nf}=W_{\infty }-W_{f}$$

Additionally, the xerogel content $X_{c}$ was determined using the $W_{\infty }$ value following Equation (6).

(6) $$X_{c}=1-W_{\infty \;}$$

Mechanical testing was performed using Zwick Retroline. Samples for compressive strength analysis were prepared from cross-linked hydrogels by cutting cylinders of ~1 cm in diameter and ~0.5 cm in height. Two samples were prepared from each hydrogel, half of which were freeze-dried and the rest were left wet. The test was carried out after precise measurement of the sample at a head speed of 1 mm/min. The samples were compressed until they reached 40% strain.

## 4. Conclusions

This study demonstrated that both the dehydration method and MXene concentration exert a decisive influence on the structural and functional properties of sodium alginate–gellan gum hydrogels. Freeze-dried samples exhibited highly porous, interconnected networks, enabling superior swelling capacity and water uptake, which are advantageous for adsorption-driven processes and tissue engineering scaffolds. In contrast, conventionally dried materials developed compact, smooth morphologies with lower porosity but enhanced barrier integrity and mechanical stability, making them more suitable for protective or load-bearing biomedical applications.

Incorporation of MXene nanosheets further modulated the hydrogel performance. At low concentrations (0.1–0.2%), MXenes reinforced the polymer matrix, improving compressive strength and elasticity. Higher contents (0.5–1.0%) reduced mechanical stability and swelling, probably due to agglomeration and excessive pore volume. This effect is beneficial especially because of the tendency of the ALG hydrogels to swell and change the dimensions and shape of the material. The ALG-GG polysaccharide blend together with the addition of the nanoadditive in form of MXene could significantly limit the unwanted ALG behavior in aqueous solutions. Thermal analyses confirmed that MXenes altered the distribution of free, bound, and non-freezing water, while also affecting thermal conductivity and stability. These results highlight that MXenes not only reinforce the hydrogel structure at low loadings but also modulate hydration dynamics and thermal response.

Overall, the findings underscore the potential of polysaccharide–MXene hydrogels as tunable biomaterials. By carefully selecting both the drying strategy and the concentration of MXene, it is possible to design composites customized for different biomedical applications, ranging from porous scaffolds for tissue regeneration to dense matrices for protective coatings or wound dressings. Future studies should focus on biological validation, including cytocompatibility, bioactivity, and long-term stability, to advance these systems toward clinical translation.

## Figures and Tables

**Table 1:** Enthalpies and phase temperatures for hydrogels after crosslinking.

MXene Content [%]	Heat of Fusion [J/g]	T_m1_ [°C]	T_m2_ [°C]	T_mo_ [°C]	T_me_ [°C]	Heat of Vaporization [J/g]	T_v_ [°C]	T_vo_ [°C]	T_ve_ [°C]
0.00%	282.14	1	8	−8	14	2049.78	108	85	122
0.10%	275.59	2	10	−6	16	2059.91	115	68	143
0.20%	254.96	1	10	−3	17	1849.68	126	80	148
0.50%	273.01	1	11	−5	16	1962.77	123	49	147
1.00%	273.40	2	10	−6	15	2004.74	101	99	133

T_m1_: melting temperature of freezing bound water; T_m2_: melting temperature of free water; T_mo_: onset temperature of melting process; T_me_: endset temperature of melting process; T_v_: evaporation temperature of water; T_vo_: onset temperature of evaporation process; T_ve_: endset temperature of evaporation process.

**Table 2:** Heats and phase temperatures for hydrogels after swelling process.

MXene Content [%]	Dehydration Type	Heat of Fusion [J/g]	T_m1_ [°C]	T_m2_ [°C]	T_mo_ [°C]	T_me_ [°C]	Heat of Vaporization [J/g]	T_v_ [°C]	T_vo_ [°C]	T_ve_ [°C]
0.00%	Lyo	285.77	3	9	−8	23	1950.20	111	33	164
0.10%	Lyo	257.80	3	11	−5	25	1816.40	119	29	160
0.20%	Lyo	278.13	3	9	−5	21	1967.28	115	29	150
0.50%	Lyo	280.59	3	10	−6	23	1970.38	107	32	144
1.00%	Lyo	283.83	3	10	−4	23	2011.83	105	29	147
0.00%	Dry	276.14	0	12	−1	20	1919.18	124	33	158
0.10%	Dry	273.04	2	11	−2	22	1951.67	120	30	151
0.20%	Dry	265.15	2	9	−6	20	1882.61	118	25	157
0.50%	Dry	270.85	2	9	−6	20	1939.25	114	28	143
1.00%	Dry	273.78	1	10	−8	22	1927.18	115	27	146

T_m1_: melting temperature of freezing bound water; T_m2_: melting temperature of free water; T_mo_: onset temperature of melting process; T_me_: endset temperature of melting process; T_v_: evaporation temperature of water; T_vo_: onset temperature of evaporation process; T_ve_: endset temperature of evaporation process.

**Table 3:** TGA results for dried swollen hydrogels and lyophilized swollen hydrogels.

MXene Content [%]	Dehydration Type	T_1%_ [°C]	T_3%_ [°C]	T_5%_ [°C]	T_10%_ [°C]	T_50%_ [°C]	T1_DTGmax_ [°C]	T2_DTGmax_ [°C]
0.00%	Lyo	36	39	42	49	83	88	257
0.10%	Lyo	38	45	50	61	107	115	253
0.20%	Lyo	36	39	42	48	79	79	258
0.50%	Lyo	36	39	43	50	86	87	256
1.00%	Lyo	35	37	39	45	73	76	255
0.00%	Dry	36	41	45	53	93	94	251
0.10%	Dry	37	41	46	55	99	101	257
0.20%	Dry	37	39	43	51	89	93	262
0.50%	Dry	35	38	42	50	88	80	259
1.00%	Dry	35	40	44	53	94	97	260

T_x%_—temperature of x% mass loss, T_DTGmax_—temperature of highest rate of mass loss (determined from DTG curves).

## Data Availability

The original contributions presented in this study are included in the article. Further inquiries can be directed to the corresponding author.

## Abbreviations

The following abbreviations are used in this manuscript:

2D	Two-Dimensional
ALG	Sodium Alginate
CaCl_2_	Calcium Chloride
CHI	Chitosan
DSC	Differential Scanning Calorimetry
EDS	Equilibrium Degree of Swelling
GG	Gellan Gum
HA	Hyaluronic Acid
HCl	Hydrochloric Acid
LiCl	Lithium Chloride
LiF	Lithium Fluoride
MILD	Minimally Intensive Layer Delamination
PVA	Polyvinyl Alcohol
SA	Sodium Alginate (used interchangeably with ALG)
SR	Swelling Ratio
SEM	Scanning Electron Microscopy
TGA	Thermogravimetric Analysis
